# Perceptions of Japanese Medical Trainees and Physicians About Writing Case Reports: A Questionnaire-Based Survey

**DOI:** 10.7759/cureus.91683

**Published:** 2025-09-05

**Authors:** Yasuhiro Kano

**Affiliations:** 1 Department of Emergency and General Medicine, Tokyo Metropolitan Tama Medical Center, Fuchu, JPN

**Keywords:** case report, internship and residency, japan, medical education, medical writing, surveys and questionnaires

## Abstract

Introduction: Writing case reports is scientifically and educationally valuable both for junior and senior physicians. To encourage this practice, the present study aimed to assess the perceptions of Japanese medical students, residents, and physicians about the value, facilitating factors, and barriers to writing case reports and specifically to compare the perceptions of trainees with those of attending physicians.

Methods: A questionnaire-based survey was administered to attendees of a lecture on case report writing held at a Japanese medical conference. The participants, who included medical students, residents, and physicians, completed a questionnaire on the benefits of, factors facilitating, and barriers to the activity of writing case reports. The respondents were categorized as trainees (postgraduate year (PGY) ≤ 6) or attending physicians (PGY ≥ 7) for comparison.

Results: In total, 116 individuals responded. There was strong consensus on the value of writing case reports, with most of the respondents agreeing with “(case report writing) improves critical thinking” (93.9% agreed/strongly agreed). “Lectures and workshops” were most often cited (98.2%) as factors facilitating the activity. The most frequently cited barriers were “lack of formal training in writing case reports” (91.4%) and “lack of training in reviewing scientific literature” (91.4%). A subgroup analysis found that the trainees were significantly more likely than the attending physicians to cite “lack of English skills” as a barrier (81.8% vs. 59.0%; p = 0.008). More attending physicians than trainees supported the mandatory presentation of case reports at conferences (p = 0.002) and setting aside time for research or other scholarly activities (p = 0.048).

Conclusions: Case reports were perceived as a highly valued academic activity among Japanese physicians in a wide range of disciplines. Identifying the respective needs of attending and early-career physicians and providing individualized support may help promote the writing of case reports.

## Introduction

With more than 25,000 being published annually in PubMed [1], case reports remain a common and fundamental format for disseminating novel clinical observations and educational insights gleaned from individual cases. The activity of writing a case report also carries a broad range of educational benefits for authors [2,3], such as improving written communication skills and critical thinking; enhancing the ability to recognize atypical presentations, which in turn sharpens observational and diagnostic skills; gaining experience in collaborative authorship and the peer-review process; deepening the understanding of patient-centered care; and fulfilling academic requirements for a degree or board certification [3-6]. For these reasons, most Japanese residents value this scholarly activity highly [7].

However, publishing a case report can be challenging for residents [7], who may lack training in reviewing scientific literature, formal instruction in writing scientifically, sufficient time away from duties to write, the financial wherewithal to engage in writing, and capable mentors [8-11]. Previous surveys on the perception about writing case reports administered to Japanese residents and physicians suffered from various limitations, such as having a small sample size, typically of less than a hundred respondents, which rendered the findings inconclusive [8,9].

Nonetheless, it remains the case that understanding the perceptions of medical students, residents, and physicians from a broad range of disciplines is necessary as a first step towards promoting the writing of case reports. The primary object of the present study involved using a questionnaire to assess the perceptions of Japanese medical students, residents (early-career physicians), and attending physicians in internal medicine about the benefits, facilitating factors, and barriers to writing case reports. The secondary objectives were to compare perceptions between trainees and attending physicians, to identify specific educational needs and training gaps, and to evaluate attitudes toward policy changes in Japan's internal medicine training system.

## Materials and methods

Study design, setting, and participants

This cross-sectional, questionnaire-based study enrolled attendees of a lecture on case report writing held during the “Koto Hajime 2025 Osaka” meeting of the Japanese Society of Internal Medicine on April 19, 2025. At the conclusion of the lecture, the attendees were invited to participate in a survey via a QR code displayed on a slide. A video recording of the lecture was made accessible on a secure, online platform from May 8 through May 31, 2025. Those who viewed the lecture online were able to participate in the survey using the same link posted on the website. The participants were asked to respond to each item in accordance with their perception or understanding of the topic prior to the lecture.

The questionnaire

The online questionnaire was created using Google Forms. No personal identifiers, such as names and email addresses, were collected. The questionnaire items, which were mainly based on previous studies to allow a comparison with their findings [10,12-14], comprised six major domains: (1) demographic characteristics (age, sex, years since graduation, and primary workplace categorized as medical school, community hospital, university hospital, clinic or others); (2) previous experience writing case reports and awareness about non-traditional case report formats, such as “clinical images”; (3) perceived benefits of writing case reports; (4) factors perceived to facilitate writing case reports; (5) perceived barriers to writing case reports; and (6) opinions about the requirements pertaining to writing case reports in Japan’s internal medicine training system. All the items in domains (3)-(6) were rated using a five-point Likert scale (“Strongly agree,” “Agree,” “Neutral,” “Disagree,” and “Strongly disagree”). The full questionnaire is provided in the Appendix.

Statistical analysis

Continuous variables were expressed as the median and interquartile range (IQR), and categorical variables were expressed as a number and percentage. The respondents were categorized by their postgraduate year (PGY) as trainees (PGY ≤ 6) or attending physicians (PGY ≥ 7) because PGY-6 is normally when physicians obtain board certification as an internist in Japan. In this study, medical students were categorized as trainees (PGY ≤ 6). Differences between the groups were assessed using the chi-square test. If an expected cell count was < 5, Fisher’s exact test was used. All the tests were two-sided, with p < 0.05 indicating statistical significance. Given the exploratory nature of this study, no adjustments were made for multiple testing. All the statistical analyses were performed using Statistical Product and Service Solutions (SPSS, version 29.0.2.0; IBM SPSS Statistics for Windows, Armonk, NY).

Ethics approval

The review board of Tokyo Metropolitan Tama Medical Center waived the requirement for an institutional review because patient data were not used. Before responding to the questionnaire, the participants were asked to consent to participation after reading a brief explanation that their anonymized responses would be used for research purposes only.

## Results

At the meeting, 181 individuals attended the lecture in person. The exact number of online (on-demand) viewers was unknown. In total, 116 individuals, consisting of 87 in-person attendees and 29 online viewers, completed the questionnaire. The response rate among in-person attendees was 48.1% (87/181); the response rate of the online viewers could not be calculated because the denominator was unknown. Table 1 shows the respondents’ baseline characteristics. The respondents were predominantly male (n=85, 73.3%). Their median age was 34 years (IQR: 26-44 years), and their median PGY was nine years (IQR: 2-20 years). The most common, primary workplace was a community hospital (n=52, 44.8%), followed by a university hospital (n=41, 35.3%). Medical students comprised 13 (11.2%) respondents. Regarding previous academic activities, 44.8% had experience publishing a study in a peer-reviewed journal, while 43.1% had experience only with giving conference presentations. Over half the respondents (n=67, 57.8%) were aware of non-traditional case report formats, such as the “clinical image.”

**Table 1:** Respondents’ baseline characteristics Note: Data are n (%) or median (IQR).

Respondents’ baseline characteristics	N=116
Age	34 (26-44)
Sex	
Male	85 (73.3)
Female	31 (26.7)
Post-graduate year (PGY)	9 (2-20)
PGY ≤ 6	55 (47.4)
PGY ≥ 7	61 (52.6)
Primary workplace	
Medical student	13 (11.2)
Community hospital	52 (44.8)
University hospital	41 (35.3)
Clinic	5 (4.3)
Others	5 (4.3)
Experience with writing case reports	
No experience (with presentations or publications)	10 (8.6)
Conference presentation experience only (no publications)	50 (43.1)
Journal submission experience only (no acceptances)	4 (3.4)
Publication experience (in a peer-reviewed journal)	52 (44.8)
Awareness of non-traditional case report formats (e.g., clinical images)	67 (57.8)

**Figure 1:**
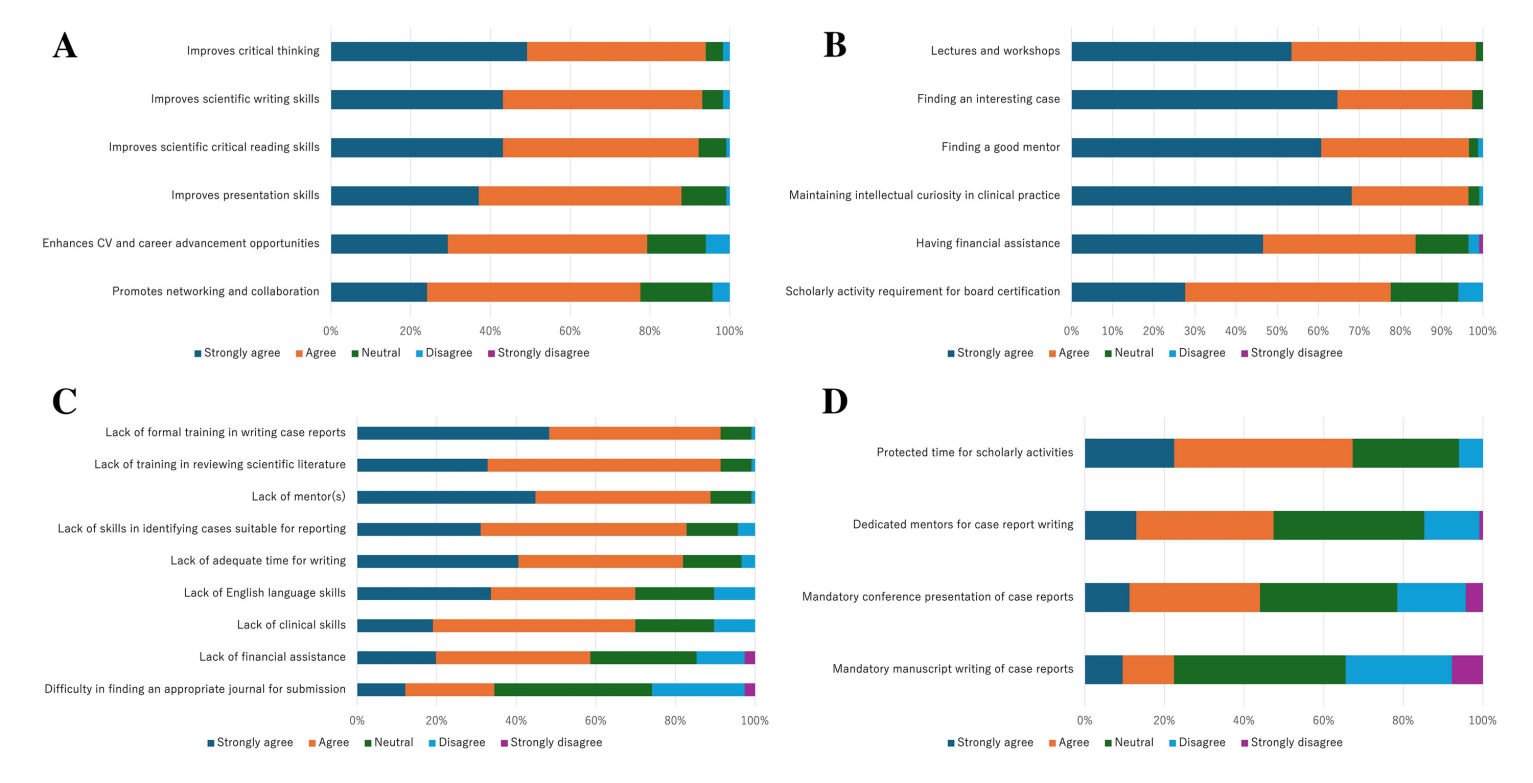
Overall perceptions of case report writing in the entire cohort (N=116) The four 100% stacked bar charts show the overall response distribution for (A) perceived benefits, (B) facilitating factors, (C) perceived barriers, and (D) opinions about the internal medicine training system. The colored segments represent the proportion of responses for each of the five items on the Likert scale (“Strongly agree,” “Agree,” “Neutral,” “Disagree,” “Strongly disagree”). For detailed, numerical data on all the items, see Table 2. Abbreviation: CV: curriculum vitae

Table 2 and Figure 1 show the respondents’ general perceptions regarding the value of, and barriers to, writing a case report. The respondents strongly endorsed writing case reports as a means of improving critical thinking (93.9% agreed or strongly agreed), scientific writing skills (93.1% agreed or strongly agreed), and scientifically critical reading skills (92.2% agreed or strongly agreed) (Figure 1A). Among the factors facilitating this activity, most of the respondents agreed that “lectures and workshops” were important (98.2% agreed or strongly agreed), along with “finding an interesting case” (97.5% agreed or strongly agreed), “finding a good mentor” (97.4% agreed or strongly agreed), and “maintaining intellectual curiosity in clinical practice” (96.5% agreed or strongly agreed) (Figure 1B). The most frequently cited barriers were “lack of formal training in writing case reports” (91.4% agreed or strongly agreed) and “lack of training in reviewing scientific literature” (91.4% agreed or strongly agreed), which were followed by “lack of mentor(s)” (88.8% agreed or strongly agreed) (Figure 1C). On the topic of the internal medicine training system (Figure 1D), 67.2% of the respondents agreed or strongly agreed that time should be set aside for research and other scholarly activities, but fewer than half supported making conference presentations, case report writing, and the provision of dedicated mentors mandatory. The fewest respondents supported “need for mandatory case report writing” (22.4%), although many respondents were undecided on the matter (43.1%).

**Table 2:** Perceptions of the value, facilitating factors, and barriers to writing case reports Abbreviation: CV: curriculum vitae

Questionnaire Items	Strongly agree	Agree	Neutral	Disagree	Strongly disagree
Benefits of writing case reports					
Improves critical thinking	57 (49.1)	52 (44.8)	5 (4.3)	2 (1.7)	0 (0.0)
Improves scientific writing skills	50 (43.1)	58 (50.0)	6 (5.2)	2 (1.7)	0 (0.0)
Improves scientific critical reading skills	50 (43.1)	57 (49.1)	8 (6.9)	1 (0.9)	0 (0.0)
Improves presentation skills	43 (37.1)	59 (50.9)	13 (11.2)	1 (0.9)	0 (0.0)
Enhances CV and career advancement opportunities	34 (29.3)	58 (50.0)	17 (14.7)	7 (6.0)	0 (0.0)
Promotes networking and collaboration	28 (24.1)	62 (53.4)	21 (18.1)	5 (4.3)	0 (0.0)
Factors facilitating writing case reports					
Lectures and workshops	62 (53.4)	52 (44.8)	2 (1.7)	0 (0.0)	0 (0.0)
Finding an interesting case	75 (64.7)	38 (32.8)	3 (2.6)	0 (0.0)	0 (0.0)
Finding a good mentor	81 (69.8)	32 (27.6)	2 (1.7)	1 (0.9)	0 (0.0)
Maintaining intellectual curiosity in clinical practice	79 (68.1)	33 (28.4)	3 (2.6)	1 (0.9)	0 (0.0)
Having financial assistance	54 (46.6)	43 (37.1)	15 (12.9)	3 (2.6)	1 (0.9)
Scholarly activity requirement for board certification	32 (27.6)	58 (50.0)	19 (16.4)	7 (6.0)	0 (0.0)
Barriers to writing case reports					
Lack of formal training in writing case reports	56 (48.3)	50 (43.1)	9 (7.8)	1 (0.9)	0 (0.0)
Lack of training in reviewing scientific literature	38 (32.8)	68 (58.6)	9 (7.8)	1 (0.9)	0 (0.0)
Lack of mentor(s)	52 (44.8)	51 (44.0)	12 (10.3)	1 (0.9)	0 (0.0)
Lack of skills in identifying cases suitable for reporting	36 (31.0)	60 (51.7)	15 (12.9)	5 (4.3)	0 (0.0)
Lack of adequate time for writing	47 (40.5)	48 (41.4)	17 (14.7)	4 (3.4)	0 (0.0)
Lack of English language skills	39 (33.6)	42 (36.2)	23 (19.8)	12 (10.3)	0 (0.0)
Lack of clinical skills	22 (19.0)	59 (50.9)	23 (19.8)	12 (10.3)	0 (0.0)
Lack of financial assistance	23 (19.8)	45 (38.8)	31 (26.7)	14 (12.1)	3 (2.6)
Difficulty in finding an appropriate journal for submission	14 (12.1)	26 (22.4)	46 (39.7)	27 (23.3)	3 (2.6)
Opinions about the internal medicine training system					
Need for protected time for scholarly activities	26 (22.4)	52 (44.8)	31 (26.7)	7 (6.0)	0 (0.0)
Need for dedicated mentors for case report writing	15 (12.9)	40 (34.5)	44 (37.9)	16 (13.8)	1 (0.9)
Need for mandatory conference presentations	13 (11.2)	38 (32.8)	40 (34.5)	20 (17.2)	5 (4.3)
Need for mandatory case report writing	11 (9.5)	15 (12.9)	50 (43.1)	31 (26.7)	9 (7.8)

**Figure 2:**
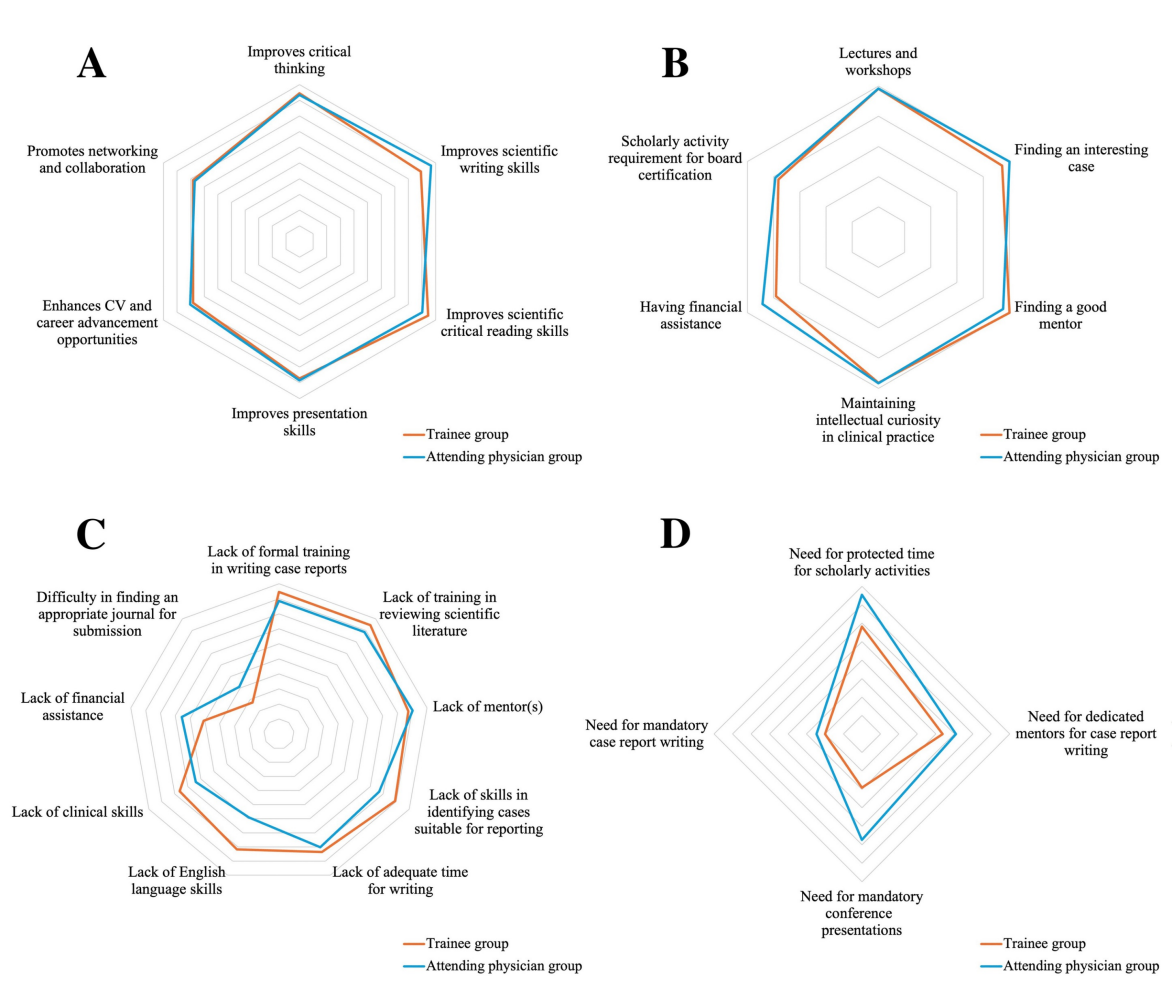
Comparison of perceptions of trainees and attending physicians about case report writing The four radar charts show perceptions about the (A) perceived benefits, (B) facilitating factors, (C) perceived barriers, and (D) the internal medicine training system. In each chart, the axes represent the percentage of positive responses (“Strongly agree” or “Agree”). The orange line indicates the trainee group (PGY ≤ 6), and the blue line indicates the attending physician group (PGY ≥ 7). For detailed, numerical data and statistical comparison of all the items, see Table 3. Abbreviation: CV: curriculum vitae

Table 3 and Figures 2A and 2B show the results of subgroup analyses comparing the proportion of participants who agreed or strongly agreed with the survey items in the trainee (PGY ≤ 6) and attending physician (PGY ≥ 7) groups. The groups did not differ significantly in terms of their perceptions of the benefits or factors facilitating case report writing. In terms of perceived barriers (Figure 2C), the trainees were significantly more likely to report “lack of English language skills” as a barrier (81.8% vs. 59.0%; p = 0.008). There were no significant intergroup differences in the other perceived barriers. Regarding the internal medicine training system (Figure 2D, Table 3), significantly fewer trainees thought that the presentation of case reports at conferences should be mandatory (29.1% vs. 57.4%; p = 0.002). However, the groups did not differ significantly in their disapproval of making case report writing mandatory (20.0% vs. 24.6%; p = 0.554). Furthermore, fewer trainees than attending physicians thought that time should be set aside specifically for the purposes of research and other scholarly pursuits (58.2% vs. 75.4%; p = 0.048).

**Table 3:** Comparison of trainees’ and attending physicians’ perceptions about case report writing ^‡^The chi‐square test or Fisher’s exact test was used to compare the trainee group (PGY ≤ 6) with the attending physician group (PGY ≥ 7). ^*^Statistically significant (p < 0.05). Abbreviation: CV: curriculum vitae

Questions	Trainee group (PGY ≤ 6, n=55)	Attending physician group (PGY ≥ 7, n=61)	p value^‡^
	Proportion of “Strongly agree” or “Agree”		
Benefits of writing case reports			
Improves critical thinking	52 (94.5)	57 (93.4)	1
Improves scientific writing skills	49 (89.1)	59 (96.7)	0.147
Improves scientific critical reading skills	52 (94.5)	55 (90.2)	0.496
Improves presentation skills	48 (87.3)	54 (88.5)	0.836
Enhances CV and career advancement opportunities	43 (78.2)	49 (80.3)	0.776
Promotes networking and collaboration	43 (78.2)	47 (77.0)	0.884
Factors facilitating writing case reports			
Lectures and workshops	54 (98.2)	60 (98.4)	1
Finding an interesting case	52 (94.5)	61 (100)	0.104
Finding a good mentor	55 (100)	58 (95.1)	0.246
Maintaining intellectual curiosity in clinical practice	53 (96.4)	59 (96.7)	1
Having financial assistance	43 (78.2)	54 (88.5)	0.133
Scholarly activity requirement for board certification	42 (76.4)	48 (78.7)	0.764
Barriers to writing case reports			
Lack of formal training in writing case reports	52 (94.5)	54 (88.5)	0.329
Lack of training in reviewing scientific literature	52 (94.5)	54 (88.5)	0.329
Lack of mentor(s)	48 (87.3)	55 (90.2)	0.622
Lack of skills in identifying cases suitable for reporting	49 (89.1)	47 (77.0)	0.086
Lack of adequate time for writing	46 (83.6)	49 (80.3)	0.644
Lack of English language skills	45 (81.8)	36 (59.0)	0.008^*^
Lack of clinical skills	42 (76.4)	39 (63.9)	0.145
Lack of financial assistance	28 (50.9)	40 (65.6)	0.109
Difficulty in finding an appropriate journal for submission	15 (27.3)	25 (41.0)	0.121
Opinions about the internal medicine training system			
Need for protected time for scholarly activities	32 (58.2)	46 (75.4)	0.048^*^
Need for dedicated mentors for case report writing	24 (43.6)	31 (50.8)	0.439
Need for mandatory conference presentations	16 (29.1)	35 (57.4)	0.002^*^

## Discussion

A key finding of this study was that both trainees and attending physicians had a positive impression of case report writing, but differed significantly in several important details. Both groups agreed that case report writing had educational benefits, such as improving critical thinking and scientific literacy. They also thought that multiple factors were important for facilitating the activity, including finding an interesting case, maintaining intellectual curiosity, and having a supportive educational system, including lectures, workshops, and good mentors. On the other hand, the trainees were more likely than attending physicians to claim that ‘lack of English language skills’ was a barrier, whereas attending physicians were more likely than trainees to endorse the idea of requiring case reports to be presented at conferences and setting aside time for research.

The general perception of case report writing as a beneficial activity, as well as the perception that having a good mentor and an interesting case were more important motivational factors than financial support, was consistent with the findings of previous studies [10,13]. In contrast, the present study differed notably from previous studies in perceptions about the role of lectures and workshops. Lectures and workshops were the least popular means of promoting case report writing (31-65%) in the previous reports, but received an approval rating of 98.2% in the present report [10,13]. Because this survey was conducted after a lecture on the topic, the very high endorsement of lectures and workshops as facilitators (98.2%) may reflect immediate post-lecture enthusiasm and not necessarily a sustained perception. However, there is also the suggestion of the possibility of a substantial, unmet educational need. The perceptions about the barriers to case report writing vary across studies, including the present one [10,13], suggesting that these barriers depend on local, institutional, or educational environments. The present study uniquely added an item pertaining to the lack of English language skills as a potential barrier, which had not been included in previous surveys [10,12-14], given the fact that most physicians in Japan do not speak English as a native language. Including this item in the present study was considered important because the lack of English language skills is a common barrier for physicians in non-native English-speaking countries [15], such as Japan. However, the findings of this study are likely to be generalizable only to nations that have a similar physician training system and a non-native English-speaking environment.

The present study found some significant differences in perceptions about case report writing between trainees and attending physicians. The former were more likely to think that their level of English language ability was a barrier to case report writing. This perception may stem from a lack of opportunities to engage with scientific literature during the early stages of their medical career, as evidenced by the fact that many trainees also lacked confidence in their ability to review scientific literature critically. However, the advent of large language models (LLMs), such as ChatGPT (OpenAI Inc., San Francisco, CA) and Gemini (Google LLC, Mountain View, CA) as “personal, round-the-clock English tutors" [16], the application of which is likely to facilitate academic writing in English by non-native English speaking professionals, has now made the language issue largely surmountable [15].

In terms of the internal medicine training system, the attending physicians more strongly supported providing financial assistance for activities related to case report writing, suggesting that this may serve as an incentive for senior staff to engage in the activity, boost their motivation to mentor their junior colleagues, and help offset the cost of publishing their research. Most trainees disapproved of making the writing and presentation of case reports at conferences mandatory. This may suggest that, while trainees acknowledge the general value of writing case reports, they also seek diverse training opportunities and object to the imposition of uniform educational requirements. However, it remains unclear why the groups differed significantly in their responses to the idea of making the writing of case reports and their presentation at conferences mandatory. One possible reason is that trainees may view publishing a paper as a more valuable academic achievement than presenting at a conference. Alternatively, because Japanese residents have more opportunities to present at conferences than to publish papers [7], they may have a more realistic perception of the burden these presentations entail.

Although this study indicates a potential need for academic support, dedicated research is necessary to establish evidence-based strategies. Nevertheless, a preliminary framework may be proposed for consideration. Initially, medical students or residency programs could integrate lectures or workshops designed to educate trainees on the significance of case reports and guide them through the writing process. Subsequently, trainees demonstrating a keen interest could be paired with a dedicated academic mentor who provides personalized guidance as distinct from clinical supervision. Finally, to ensure sustainability, institutions may provide tangible support, especially financial incentives to mentors and subsidies for language support services, including professional English editing. Validated descriptive or interventional studies are needed to verify the appropriateness and effectiveness of such proposals. Moreover, the present study is limited to case report writing; thus, support strategies for other types of research may differ.

The present study has several limitations. First, there may have been a selection bias in the choice of the participants, as they were recruited from attendees at a lecture delivered during an academic conference on internal medicine and may therefore have already had a strong interest in writing case reports. Furthermore, the response rate of the participants who were recruited online could not be calculated. Thus, studies enrolling various other groups in the medical profession are warranted to assess the generalizability of the present findings. As such findings accumulate in the future, the differences in cognitive tendencies among various populations will likely become clearer. Second, the sample size was relatively small. However, it is noteworthy that the sample pool in the present study is the largest among similar studies in Japan to date [8,9]. Third, the questionnaire relied exclusively on closed-ended items that were based on previous studies and did not include open-ended questions; thus, the full spectrum of the participants’ perceptions may not have been captured. Furthermore, although the questionnaire items used in this study were designed primarily on the basis of previous findings, their psychometric validity has yet to be established. While the use of the multiple-choice format was intended to improve the response rate by prioritizing the ease of responding, future exploratory studies incorporating qualitative feedback are warranted to gather a broader range of opinions. Finally, this study explored participants’ current perceptions, which do not necessarily reflect their actual experience of writing case reports. Future studies stratifying participants by their actual experience in writing case reports would provide more empirical evidence.

## Conclusions

In conclusion, this study reaffirmed the likelihood that case reports were regarded by Japanese medical students, residents, and physicians at various stages in their careers as a valuable academic activity. Perceptions differed between attending and early-career physicians regarding the barrier of lack of English language skills and, within the training system, the need for protected scholarly time and for mandatory conference presentations. Identifying the respective needs of attending and early-career physicians and providing individualized support may help promote the writing of case reports.

## Appendices

Appendix: Survey questionnaire items used in the study (translated into English from Japanese)

A. Demographics

1. Age in years (number)

2. Sex (Male / Female)

3. Years since graduation from medical school (number, enter 0 if you are a medical student)

4. Current status/work place (Medical student / Community hospital / University hospital / Clinic / Other)

B. Experience with writing case reports

1. Please indicate your academic experience with writing case reports to date. Please report your experience as a first author rather than as a co‑presenter or co‑author.

(I have presented a case report at a conference but I have never written a case report. / I submitted a case report to a peer‑reviewed medical journal, but it was not accepted. / I have submitted a case report manuscript to a peer‑reviewed medical journal, and it was accepted. / I have neither presented a case report at a conference nor written one.)

2. Were you aware that case reports can have formats (e.g., clinical image articles) other than the traditional one? (Yes / No)

C. Perceptions about the benefits of writing case reports

Please indicate whether you consider each of the items below to be a benefit of writing case reports. Response options (5‑point Likert scale for all items in this section): Strongly agree / Agree / Neutral / Disagree / Strongly disagree.

1. Improves scientific writing skills.

2. Enhances curriculum vitae and career advancement opportunities.

3. Improves presentation skills.

4. Improves critical thinking.

5. Improves scientific critical reading skills.

6. Promotes networking and collaboration opportunities.

D. Perceptions about factors facilitating case report writing

Please indicate whether you consider each of the items below to be a factor facilitating case reports writing. Response options (5‑point Likert scale for all items in this section): Strongly agree / Agree / Neutral / Disagree / Strongly disagree.

1. Finding a good mentor promotes writing case reports.

2. Finding an interesting case promotes writing case reports.

3. Maintaining intellectual curiosity in clinical practice promotes writing case reports.

4. The availability of financial support for scholarly activities promotes the presentation and writing of case reports.

5. Lectures and workshops on case report writing promote writing case reports.

6. A scholarly activity requirement for board certification promotes writing case reports.

E. Perceptions about barriers to case report writing

Please indicate whether you consider each of the items below to be a barrier to case report writing.

Response options (5‑point Likert scale for all items in this section):

Strongly agree / Agree / Neutral / Disagree / Strongly disagree

1. Lack of clinical skills.

2. Lack of skills in identifying cases suitable for reporting.

3. Lack of formal training in writing case reports.

4. Lack of English language skills.

5. Lack of mentor(s) who can support case report writing.

6. Lack of adequate time for writing.

7. Lack of training in reviewing scientific literature.

8. Insufficient financial support for scholarly activities.

9. Difficulty in finding an appropriate journal for submission.

F. Opinions about the internal medicine training system

For each statement below, please indicate your level of agreement.

Response options (5‑point Likert scale for all items in this section):

Strongly agree / Agree / Neutral / Disagree / Strongly disagree

1. Case report presentations at academic meetings should be mandatory during residency.

2. Writing a case report should be mandatory during residency.

3. There should be mentors dedicated specifically to supporting case report writing.

4. Time should be set aside for scholarly activities.
